# Determination of Iron Valence States Around Pits and the Influence of Fe^3+^ on the Pitting Corrosion of 304 Stainless Steel

**DOI:** 10.3390/ma13030726

**Published:** 2020-02-05

**Authors:** Hao Zhang, Nan Du, Shuaixing Wang, Qing Zhao, Wenjie Zhou

**Affiliations:** National Defense Key Discipline Laboratory of Light Alloy Processing Science and Technology, Nanchang Hangkong University, Nanchang 330063, China; zhanghhao6666@126.com (H.Z.); z_haoqing@sina.com (Q.Z.); wanan12w@163.com (W.Z.)

**Keywords:** stainless steel, pitting corrosion, chromogenic method, Fe^3+^ reduction

## Abstract

Potassium ferricyanide and potassium ferrocyanide were used to observe and monitor the pitting corrosion of 304 stainless steel (SS) at anodic polarization in situ. The results show that there are Fe^3+^ ions around the corrosion pit when pitting occurs on 304 SS in NaCl aqueous solution. The effect of Fe^3+^ surrounded pits on the pitting corrosion was also studied by testing the electrochemical behavior of 304 SS in different Fe^3+^/Fe^2+^ solutions. The presence of Fe^3+^ leads to the positive shift of corrosion potential and the increase of corrosion rate of 304 SS. There are two possible reasons for this phenomenon. On the one hand, Fe^3+^ hydrolysis results in the decrease of pH value of solution. At the same iron ion concentration, the higher the Fe^3+^ ion concentration, the lower the solution pH value. On the other hand, Fe^3+^ may reduce on the electrode surface. The decrease of solution pH and the reduction of Fe^3+^ resulted in the acceleration of the corrosion rate.

## 1. Introduction

Pitting corrosion is a common localized corrosion form of stainless steel (SS) in chloride-containing environments [1,2,3,4]. Pitting is generally focused on a small area of a metal surface and causes devices to fail by perforation or initiates stress corrosion cracks [5,6]. It is widely accepted that oxygen reduction and/or hydrogen reduction reactions occur at the cathode during the corrosion of metals [7,8,9], but for the pitting corrosion of stainless steel, the cathodic reaction becomes very complex.

It is known that some corrosion products diffuse to the outside of pits during the growth of corrosion pits [10,11]. The hydrolysis of cations changes the corrosive environment of the passive film outside the pits [12]. Fe^2+^ is the main corrosion product in the pitting process of 304 SS, but it is easily oxidized to Fe^3+^ [13,14] in electrolytes containing oxidants such as Equation (1).

(1) $$2{\mathrm{Fe}}^{2+}+\frac{1}{2}O_{2}+\;2H^{+}=\;2{\mathrm{Fe}}^{3+}+H_{2}O$$

Meanwhile, the use of FeCl_3_ solution as a corrosion medium to study the pitting corrosion behavior of SS has been widely accepted [15,16]. However, as Fe^3+^ reduction may occur on the cathode in FeCl_3_ solution, and it is unknown whether Fe^3+^ exists around the pit and whether Fe^3+^ reduction reaction will occur on the surface of SS during pitting corrosion, so it is not accurate to use FeCl_3_ as corrosion medium to study pitting corrosion of stainless steel.

Fe^3+^ cannot exist stably on the surface of electrodes because the surface potential of 304 SS electrodes in an open-circuit state is lower than that of Fe^3+^ reduction [9]. This raises a new problem regarding whether there are no Fe^3+^ ions around the corrosion pit of SS or there are Fe^3+^ ions, but they are reduced on the electrode surface. Oxides of Fe(III), such as α-FeOOH, γ-FeOOH and Fe_3_O_4_, are founded in the corrosion products of SS or iron [8,17,18,19], but there is no relevant report to confirm the existence of Fe^3+^ ions around the pits for the pitting corrosion of SS.

Researchers have found that Fe^2+^ and Fe^3+^ can be sensitively detected by potassium ferricyanide [20,21] and potassium ferrocyanide [22,23], respectively. Therefore, potassium ferricyanide and potassium ferrocyanide are used to verify the presence of Fe^3+^ around the pits of SS in this work. The electrochemical behavior of 304 SS in a 0.1 mol/L iron ion solution with different Fe^3+^/Fe^2+^ ratios was also studied using potentiodynamic polarization curves. Based on this, the effect of Fe^3+^ surrounded pits on the pitting corrosion of 304 SS was discussed. 

## 2. Experimental

### 2.1. Sample Preparation

Type 304 SS, with a chemical composition (wt.%) of 0.035 C, 0.52 Si, 1.18 Mn, 0.036 P, 0.026 S, 18.21 Cr, 8.03 Ni and balanced with Fe, was used as the specimen in this work. The 304 SS was cut into 1 mm and 10 mm diameter cylinders. These samples were soldered to copper wires by tin and then embedded in epoxy resin to act as the working electrode (WE). All the samples were finished by wet-grinding with a series of emery papers from 400 to 1200 grit, cleaned with distilled water and acetone, and dried in cold air.

### 2.2. Chromogenic Testing

**Figure 1:**
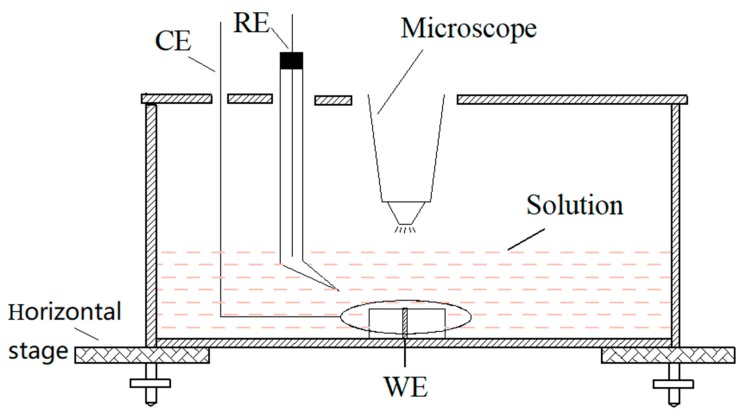
The schematic of chromogenic testing device.

The distribution and diffusion of corrosion products during the pitting corrosion of 304 SS were observed with the experimental device shown in Figure 1. Potassium ferricyanide and potassium ferrocyanide was used to observe and to monitor the pitting corrosion of 304 stainless steel (SS) at anodic polarization in situ. A KH-7700 three-dimensional video microscope (Hirox Co., Ltd., Tokyo, Japan) lens was placed above the sample to monitor the surface morphology of sample in real-time. During the chromogenic testing, the 304 SS electrode with a diameter of 1 mm or 10 mm was potentiostatically polarized in a 3.5 wt.% NaCl and 0.05 wt.% K_3_[Fe(CN)_6_] solution or a 3.5 wt.% NaCl and 0.05 wt.% K_4_[Fe(CN)_6_]·3H_2_O solution, respectively. In addition, neither of the two solutions were oxygenated or deaerated. In order to obtain the growth parameters of a single pit, it was necessary to use an electrode with a 1 mm diameter (area of 7.85 × 10^−3^ cm^2^), whereas a 10-mm diameter electrode was used to obtain the statistical data of a large number of stable pits.

The chrono amperometry curve was carried out on an Autolab PGSTAT 302N electrochemical workstation (Metrohm A G, Herisau, Switzerland), a saturated calomel electrode (SCE) was used as the reference electrode (RE) and a platinum sheet around the sample was used as the counter electrode (CE), the applied potential was 0.35 V (SCE). Prior to testing, the electrodes were cathodically polarized for 20 min at –1.2 V(SCE) to remove the oxide film on the surface and were then immersed in a 3.5% NaCl solution for 3 h to reconstitute a stable passive film [24]. According to the literature [20,21,22,23], K_3_[Fe(CN)_6_] and K_4_[Fe(CN)_6_]·3H_2_O could react with Fe^2+^ and Fe^3+^, respectively, to generate Prussian blue and then to detect the presence of Fe^2+^ and Fe^3+^ in the solution.

### 2.3. Electrochemical Testing of 304 SS in Different Iron Ion Solutions

The effect of Fe^3+^-surrounded pits on the pitting corrosion of 304 SS was studied by testing the electrochemical behavior of 304 SS in different iron ion solutions. According to the results of chromogenic testing, iron ions outside the pits can be maintained at a concentration of 0.36 mol/L or higher during the growth of the pits. In this work, 0.1 mol/L iron ion solutions were selected as the testing solution, as shown in Table 1. as shown in Table 1. Deionized water was poured into a flask and bubbled with argon for 30 min. FeCl_3_·6H_2_O and FeCl_2_·4H_2_O were then added for the preparation of iron ion solutions with different Fe^3+^/Fe^2+^ ratios. The pH value of the solution was measured by a pH meter (METTLER TOLEDO FE20, Mettler-Toledo, Zurich, Switzerland) before testing. 

Subsequently, type 304 SS samples with diameters of 10 mm were used as the working electrodes in these tests. Prior to testing, the electrodes were cathodically polarized for 20 min at −1.2 V (vs. SCE) to remove the oxide film on the surface and were then immersed in a 3.5% NaCl solution for 3 h to reconstitute a stable passive film [24,25]. Potentiodynamic polarization measurement was carried out at a potential scanning rate of 2 mV/s. In addition, all tests were repeated using three duplicate specimens to confirm reproducibility.

## 3. Results and Discussion

### 3.1. Chromogenic Testing Results

**Figure 2:**
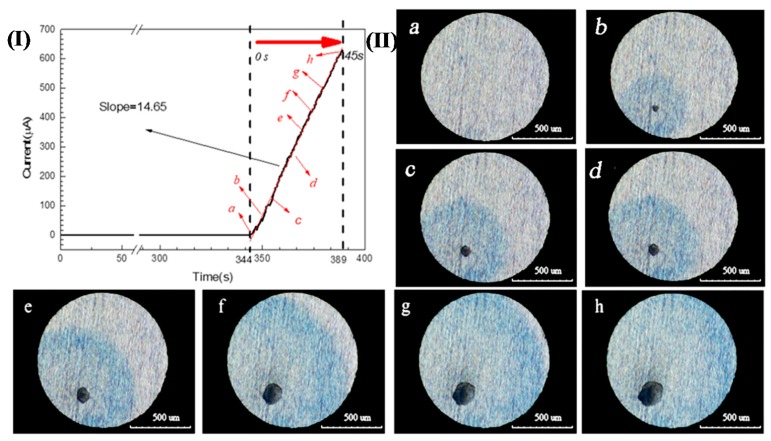
(**I**). Chrono amperometry curve of 304 SS in a 3.5 wt.% NaCl and 0.05 wt.% K_3_[Fe(CN)_6_] solution at 0.35 V(SCE); (**II**). Images of the growth of a single pit over time corresponding to Figure 2(I): (**a**) 0 s, (**b**) 5 s, (**c**) 10 s, (**d**) 20s, (**e**) 30 s, (**f**) 35 s, (**g**) 40 s, and (**h**) 45 s.

A 304 SS electrode with a diameter of 1 mm was potentiostatically polarized in a 3.5 wt.% NaCl and 0.05 wt.% K_3_[Fe(CN)_6_] solution (this solution is not oxygenated/deaerated) at 0.35 V (SCE) for 400 s. The corresponding current-time curve is shown in Figure 2(I). The corrosion current increased sharply at 344 s, which is a typical sign that pitting corrosion occurred on the sample surface [3,5,20,21]. Meanwhile, the corrosion pits and blue spots could be observed by the three-dimensional microscope. If this time was redefined as 0 s, the growth of a single pit with time is shown in Figure 2(II)a–h. It can be seen that the current value increased continuously and the size of the pit mouth and blue spot continued to expand as the polarization progressed. Fe^2+^ was the only substance in the solution that can make potassium ferricyanide blue, so it can be confirmed that Fe^2+^ was present around pitting holes of 304SS.

**Figure 3:**
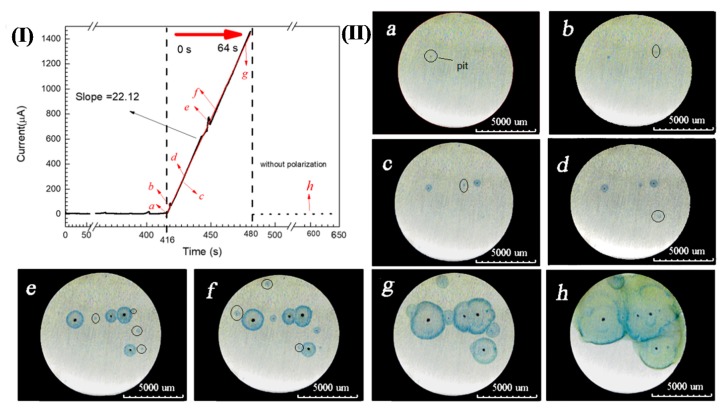
(**I**) Chrono amperometry curve of 304 SS in a 3.5 wt.% NaCl and 0.05 wt.% K_4_[Fe(CN)_6_]·3H_2_O solution at 0.35 V(SCE). (**II**). Distribution and diffusion of pitting corrosion products over time corresponding to Figure 3(I): (**a**) 0 s, (**b**) 2 s, (**c**) 11 s, (**d**) 13 s, (**e**) 33 s, (**f**) 38 s, (**g**) 61 s and (**h**) 184 s.

In addition, the presence of Fe^3+^ during pitting corrosion was detected by the chromogenic reaction when 304 SS was potentiostatically polarized in a 3.5 wt.% NaCl and 0.05 wt.% K_4_[Fe(CN)_6_]·3H_2_O solution at 0.35 V (SCE), as shown in Figure 3(I). Figure 3(II)a–h clearly show that blue spots appeared around the corrosion pits and the size of blue spots also expanded with the growth of corrosion pits. Since the electrolyte did not contain K_3_[Fe(CN)_6_] and there were no other ions that can made K_4_[Fe(CN)_6_]·3H_2_O discolor, so this might be the color reaction of Fe^3+^ and K_4_[Fe(CN)_6_]·3H_2_O in the solution. This chromogenic phenomenon fully indicated that both Fe^2+^ and Fe^3+^ are present in the corrosion products around the corrosion pits of 304 SS.

**Figure 4:**
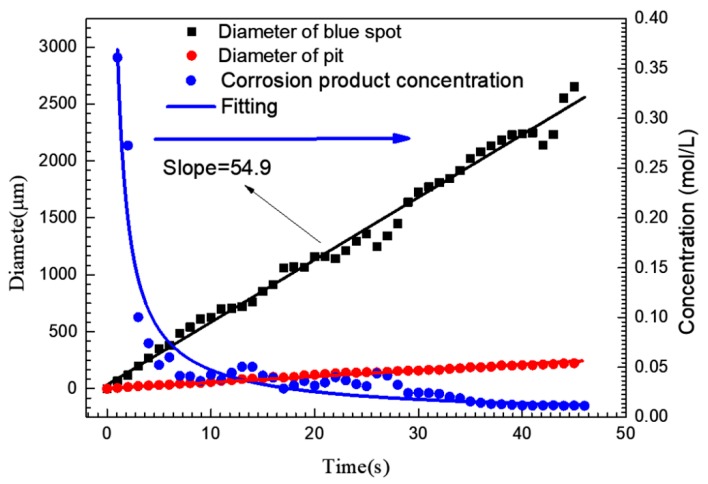
Variation curve of pit mouth diameter, blue spot diameter and the average corrosion product concentration in the blue spot area corresponding to a single pit corresponding to Figure 2(I).

The blue spot and pit diameters shown in Figure 2 are plotted in Figure 4. The average concentration of iron ions around the pit could be approximately calculated from this. According to the current-time (*i-t*) curve shown in Figure 2(I), the amount of charge (*Q*) generated by the growth of the pit could be obtained by integrating *i* with *t*. The total molar amount of iron ions (including ferric and ferrous) produced by the growth of pit could also be calculated by Faraday’s law [6], so Equations (2) and (3) are applicable:

(2) Q=∫idt=zCFV

(3) $$C=\frac{\int idt}{zFV}$$

where *z* is the average cation valence (2.19) [6], *F* is the Faraday constant (96,500 C/mol) and *V* is the volume of the blue spot, C is the concentration of iron ions (the elements such as Cr and Ni were ignored). If the blue spot was assumed to be hemispherical and all corrosion products diffused into the blue spot, the average concentration of iron ions in the blue spot at different times was calculated, as shown in Figure 4. At the initial stage of pit growth, the iron ion concentration in the blue spot could reach 0.36 mol/L. When the pit grew to 3 s, the average concentration of iron ions in the blue spot decreased dramatically to 0.1 mol/L, the diameter of blue spot was up to 200 μm at this time, while the diameter of the pit mouth was only 23 μm.

If the convection and electromigration in the process of corrosion product migration are ignored and only the diffusion process is considered, the iron ion concentration in the center of the blue point can be estimated roughly by Fick’s second law.

(4) $$c\left(x,t\right)=c_{0}+\left(c_{s}-c_{0}\right)\left[1-erf\left(\frac{x}{2\sqrt{Dt}}\right)\right]$$

where *c* is the concentration of corrosion product (iron ions), *x* is the distance from the center point, *t* is the diffusion time of corrosion products, *c*_0_ is the concentration of iron ions (including ferric and ferrous) in the bulk solution and *c_center_* is the iron ion concentration at the center of blue spot. According to Figure 4, the diameter of the blue spot grew linearly with the polarization time. Hence, the concentration of iron ions at the blue point boundary (*c_b_*) can be expressed as:

(5) $$c_{b}=c\left(vt,t\right)=c_{0}+\left(c_{center}-c_{0}\right)\left[1-erf\left(\frac{vt}{2\sqrt{Dt}}\right)\right]$$

where *v* is the horizontal diffusion velocity of the blue spot. The concentration of iron ions at the center of the blue point can be expressed as:

(6) $$c_{center}=c_{0}+\left[\frac{c_{b}-c_{0}}{1-erf\left(\frac{v\sqrt{t}}{2\sqrt{D}}\right)}\right]$$

Due to *c*_0_, *c_b_*, *D,* and *v* being constants, it can be seen from Equation (5) that the iron ion concentration at the center of the blue point (*c_center_*) is positively correlated with the pit growth time, that is to say, for the single pit given in Figure 2, the concentration of iron ions at the orifice increases with the growth of the pit.

On the basis of the above, iron ions outside the pits can be maintained at a concentration of 0.36 mol/L or higher during the growth of the pits. In this work, 0.1 mol/L iron ion solutions were selected as the research object to discuss the effect of the transformation from Fe^2+^ to Fe^3+^ on the pitting corrosion of 304 SS by changing the Fe^2+^/Fe^3+^ ratio. 

### 3.2. Electrochemical Testing Results of 304 SS in Different Iron Ion Solutions

**Figure 5:**
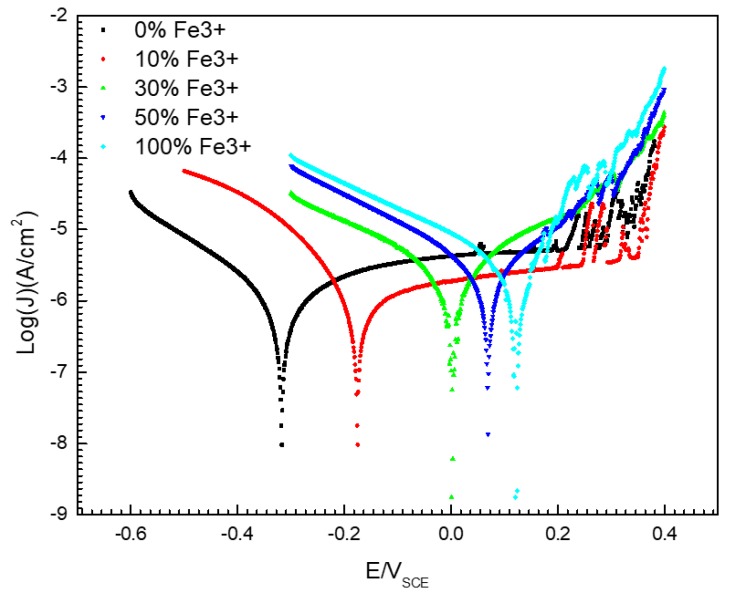
Potentiodynamic polarization curves of 304 SS in 0.1 mol/L iron ion solutions (deaerated) with different proportions of Fe^2+^/Fe^3+^.

Figure 5 gives the potentiodynamic polarization curves of 304 SS in a 0.1 mol/L iron ion solution (deaerated) with different concentrations of Fe^3+^. It is shown that there was a significant passive zone in the anode branch of the polarization curve for 304 SS in the 0.1 mol/L FeCl_2_ solution without Fe^3+^. The passivation range is between −0.3–0.3 V (SEC), but the current density fluctuated between 0.2–0.3 V (SEC), this is because the passivation film on the surface of 304 SS is unstable when the potential is between 0.2–0.3 V (SEC), and metastable pits are easy to appear on the surface. The constant initiation and repassivation of pits lead to current peaks in the anode polarization curve. However, if 30% Fe^2+^ (or more) in solution was replaced by Fe^3+^, the steel showed active dissolution behavior. Moreover, the corrosion potential of the 304 SS shifted positively and the corrosion current density increased with increasing Fe^3+^ content. As shown in Figure 5 and Table 2, the corrosion potential of 304 SS in the 0.1 mol/L FeCl_2_ solution was −0.316 V (SCE), but the value in the 0.1 mol/L FeCl_3_ solution was 0.123 V (SCE). The corrosion potential of 304 SS in the 0.1 mol/L FeCl_3_ solution is shifted positively by about 0.439 V (SCE) more than that in the 0.1 mol/L FeCl_2_ solution. At the same time, the corrosion current density (*J_corr_*) increased from 1.09 μA/cm^2^ to 3.42 μA/cm^2^.

**Figure 6:**
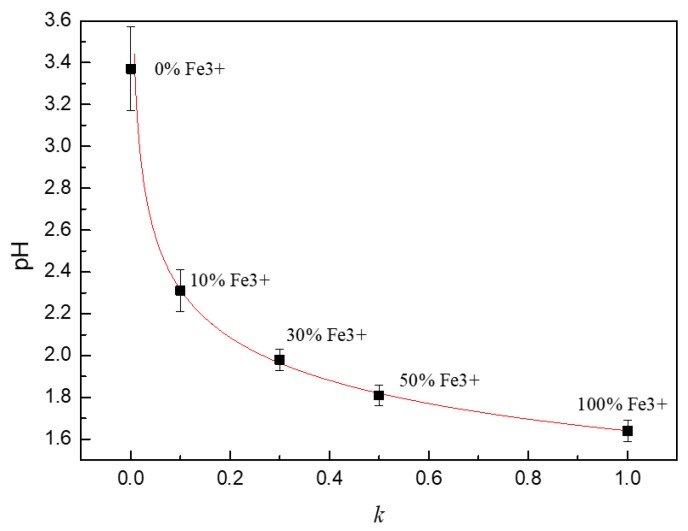
The pH value of solutions with different proportions of Fe^2+^/Fe^3+^.

The pH value of the solution with different proportions of Fe^2+^/Fe^3+^ is shown in Figure 6. It can be seen that the pH value of the 0.1 mol/L FeCl_2_ solution without Fe^3+^ was 3.37. The pH value of the solution rapidly decreased to 2.31 when 10% Fe^2+^ was replaced by Fe^3+^ and the concentration of free hydrogen ions increased by 11.5 times. If all the Fe^2+^ in the solution was completely replaced by Fe^3+^, the pH value of the solution decreased to 1.64.

The pH value of solution decreased significantly with the increase of Fe^3+^ in the simulated solution, which is because the hydrolysis ability of Fe^3+^ iron is much greater than that of Fe^2+^ iron. The solubility product constants of Fe(OH)_2_ and Fe(OH)_3_ are quite different according to Lange’s handbook of chemistry. The solubility product constant of Fe(OH)_2_ is 4.87 × 10^−17^,and the solubility product constant of Fe(OH)_3_ is 2.79 × 10^−39^. Assuming that the concentration of Fe^2+^ around the pit is 0.1 mol/L, which can be obtained according to the definition of stable product:

(7) $$\left(0.1-a_{Fe^{2+}}\right){\left(\frac{10^{-14}}{2a_{Fe^{2+}}+10^{-7}}\right)}^{2}=4.87\times 10^{-17}$$

(8) $$\mathrm{pH}=-\mathrm{lg}(2a_{Fe^{2+}}+10^{-7})$$

Assuming that Fe^2+^ completely transforms Fe^3+^, we can get:

(9) $$\left(0.1-a_{Fe^{3+}}\right){\left(\frac{10^{-14}}{2a_{Fe^{3+}}+10^{-7}}\right)}^{3}=2.79\times 10^{-39}$$

(10) $$\mathrm{pH}=-\mathrm{lg}(2a_{Fe^{3+}}+10^{-7})$$

The calculation results shown that the hydrolysis molar quantity of Fe^2+^($a_{Fe^{2+}}$) is about 6.65 × 10^−7^ mol/L and the hydrolysis molar quantity of Fe^3+^ is about 0.0156 mol/L. The hydrolysis ability of Fe^3+^ is much greater than that of Fe^2+^.

Some research shows that the decrease of pH value will lead to the positive shift of corrosion potential and the increase of corrosion rate of 304 SS [8,26,27]. The reason is that in acidic solutions, especially when the pH is low, the hydrogen evolution reaction accounts for a large proportion in the cathode process. With the decrease of pH, the rate of hydrogen evolution increases, which consumes the electrons on the electrode surface and leads to the rise of potential. It seems reasonable to explain the experiment from this point of view, but there is an experimental phenomenon that cannot be ignored, that is, the corrosion potential is too positive of 304 SS in the solution with the presence of Fe^3+^.

### 3.3. Analysis of Thermodynamic Conditions

It is widely accepted that oxygen reduction and/or hydrogen reduction reactions occur at the cathode during the pitting corrosion of SS [7,8,9]. The equilibrium electrode potentials of the two reactions are calculated according to the Nernst equation (Equations (11)–(13)).

(11) $$E^{e}=E^{\theta }+\frac{2.3RT}{nF}\mathrm{Log}\frac{\left(a_{A}^{a}\right)}{\left(a_{B}^{b}\right)}$$

(12) $$E_{O}^{e}=1.229-0.0591\mathrm{pH}$$

(13) $$E_{H}^{e}=-0.0591\mathrm{pH}$$

where *E^e^* is the equilibrium electrode potential, *E^θ^* is the standard electrode potential, *R* is the constant of molar gas, *T* is the thermodynamic temperature (298 K), *n* is the number of electrons participating in the reaction, *F* is the Faraday constant, *a_A_* and *a_B_* are the concentration of oxidized and reduced substances, and *a* and *b* are the stoichiometric coefficient of substances A and B, respectively. $E_{O}^{e}$ and $E_{H}^{e}$ are the equilibrium electrode potential of oxygen reduction and hydrogen reduction, respectively. According to the pH value of the solution shown in Figure 6 and Equations (12) and (13), the equilibrium electrode potential of oxygen and hydrogen reduction is shown in Table 3.

Figure 5 and Table 3 show the corrosion potential of 304 SS in solutions with different proportions of Fe^2+^/Fe^3+^. It is clear that the self-corrosion potential of 304 SS is more positive than the equilibrium electrode potential of hydrogen reduction in all testing solutions, which means that there is no possibility of hydrogen reduction for 304 SS in 0.1 mol/L iron ion solutions.

Hydrogen or/and oxygen reduction reactions occur during the iron or steel anodic dissolution process in the light of the different of pH values and dissolved oxygen concentrations. The pH conditions for hydrogen reduction may be different, but in any case, the thermodynamic conditions of hydrogen evolution can be reached with the decrease of pH value [8,26,27]. However, in the solution containing Fe^3+^, the corrosion potential of 304 SS is too positive to meet the thermodynamic conditions of the hydrogen reaction.

### 3.4. Cathodic Reaction

The results of the thermodynamic analysis show that no hydrogen reaction occurs for 304 SS in 0.1 mol/L iron ion solutions. The decrease in pH value is not the main reason for the positive shift of corrosion potential and the increase of corrosion rate for 304 SS in the 0.1 mol/L iron ion solution with different proportions of Fe^2+^/Fe^3+^. Considering the positive shift of the corrosion potential with the increase of Fe^3+^ content, we hold that the presence of Fe^3+^ might change the cathodic reaction of pit corrosion for SS. In the corrosive environment containing Fe^3+^, the reduction reaction of Fe^3+^ (Equation (14)) might occur on the electrode surface:

(14) $$Fe^{3+}+e↔Fe^{2+}$$

(15) $$E_{Fe^{2+}/Fe^{3+}}^{e}=0.771+0.0591\mathrm{lg}a_{Fe^{3+}}/a_{Fe^{2+}}-0.2438$$

The equilibrium electrode potentials of the reduction reaction of Fe^3+^ ($E_{Fe^{2+}/Fe^{3+}}^{e}$) were calculated according to the Nernst equation (Equations (11) and (15)) (assuming that the concentration of Fe^3+^ in the 0.1 mol/L FeCl_2_ solution and the concentration of Fe^2+^ in the 0.1 mol/L FeCl_3_ solution is 10^−6^ mol/L). The calculation results are shown in Table 3.

**Figure 7:**
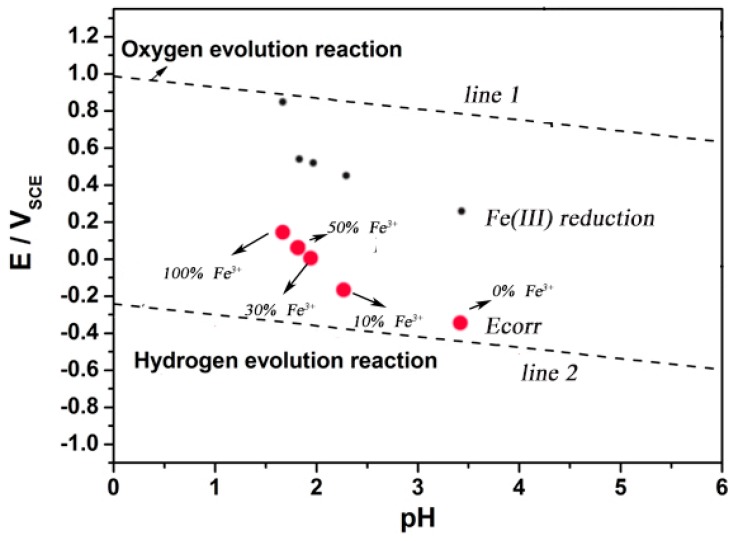
An experimental E-pH diagram of 304 stainless steel at pH varying from 0 to 6.

The results show that the corrosion potential of 304 SS in the solution containing Fe^3+^ satisfied the thermodynamic conditions of Fe^3+^ reduction. It was possible for the reduction reaction of Fe^3+^ on the surface of 304 SS. Although the proportion of Fe^3+^ reduction in all cathodic reactions was unknown, the reduction of Fe^3+^ might be an important reason for accelerating corrosion of SS. As shown in Figure 7, obviously, of the five solutions used in this test, the self-corrosion potential of 304 SS is higher than the thermodynamic results based on the hydrogen evolution reaction and more negatively than that of the oxygen evolution reaction and Fe(III) reduction reaction. The test solution used in the experiment was subjected to argon deoxidation treatment. Therefore, it is reasonable to think that the reduction reaction of Fe^3+^ in the solution on the electrode surface will lead to the positive shift of self-corrosion potential and the increase of corrosion rate of SS.

### 3.5. Effect of Fe^3+^ Around the Pit on the Pitting Corrosion of 304 SS

When a corrosion pit appears on the surface of SS, the corrosion products migrate to the outside of the pit, as shown in Figure 2. Under the action of oxidizing substances, such as dissolved oxygen, Fe^2+^ in corrosion products outside the pits can be converted into Fe^3+^, which leads to the appearance of blue spots in the potassium ferrocyanide solution, as shown in Figure 3. This phenomenon confirms that there are Fe^3+^ ions around the corrosion pit, which is of great significance to understand the pitting mechanism of SS. On the one hand, the hydrolysis of Fe^3+^ results in the decrease of the pH of the solution around the pits. As shown in Figure 6, the pH value of the 0.1 mol/L FeCl_3_ solution is 1.64 and the value of the 0.1 mol/L FeCl_2_ solution is only 3.37. Although as for the hydrolysis of corrosion products for SS, the hydrolysis of Cr^3+^ and Ni^2+^ plays a dominant role, but the presence of Fe^3+^ makes the corrosive environment more acidic. As the pH of the solution decreased, the corrosion of the SS accelerated and the passive film was more vulnerable to damage [8,26,28]. On the other hand, the presence of Fe^3+^ might change the cathodic reaction of pit corrosion for SS. As shown in Figure 5 and Figure 7, due to the existence of Fe^3+^, the corrosion potential of 304 SS is shifted positively, which makes the hydrogen evolution in low pH environments become difficult. This includes the 0.1 mol/L FeCl_3_ solution, where the corrosion potential of 304 SS is 0.123 V (SCE), which is much higher than the thermodynamic results based on hydrogen reduction. At this time, Fe^3+^ reduction may be the main cathodic reaction on the surface of SS.

It has been reported that when SS pitting occurs, the anode reaction is mainly carried out in the etched hole, while the cathode reaction is mainly carried out outside the etched hole [28,29,30]. That is to say, the cathode reaction outside the hole has an important influence on the growth of the pits. Due to the existence of a diffusion barrier, the corrosion products inside the etched pit are difficult to diffuse outwards, and the oxidizing substances outside the pit are also difficult to diffuse into the pit, and there may be no Fe^3+^ ions in the hole. However, dissolved oxygen can migrate from the bulk solution to the sample surface without restriction, Fe^2+^ ions can be oxidized to Fe^3+^ ions outside the pit, and Figure 3 proves that there are Fe^3+^ ions around the corrosion pit.

**Figure 8:**
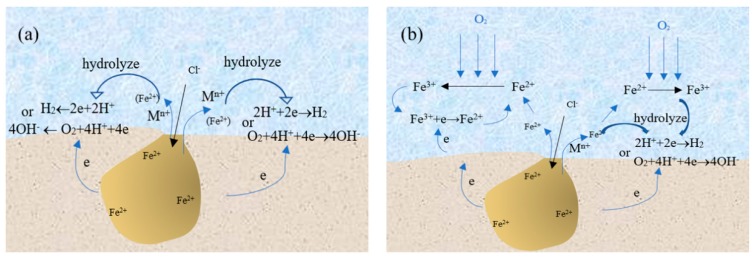
Pitting corrosion model for stainless steel in the environment without (**a**) and with (**b**) transformation from Fe^2+^ to Fe^3+^.

In summary, the presence of Fe^3+^ around the pits has an important influence on the pitting corrosion behavior of SS. If there is no Fe^3+^ around the pits, the growth mechanism of pitting corrosion can be explained by Figure 8a. The corrosion products of SS migrate to the outside of the pits and the hydrolysis of the corrosion products around the pits results in a decrease of pH for the solution around them. At the same time, the hydrogen or oxygen reduction reaction occurs around the pits, which promotes the growth of the corrosion pit. However, in this work, the experimental results show that there are Fe^3+^ around the pits. If Fe^3+^ was derived from the oxidation of Fe^2+^ under the action of oxidizing substances, the growth mechanism of pitting corrosion of 304 SS could be shown in Figure 8b. Some Fe^2+^ ions migrating from the pits will be directly hydrolyzed around the pits while others are converted into Fe^3+^ by the action of the oxidizing substance. On the one hand, the pH value of solution was lower at the same concentration of iron ions due to the strong hydrolysis ability of Fe^3+^. On the other hand, Fe^3+^ can undergo the reduction reaction to consume electrons and promote pit growth.

## 4. Conclusions

1. Potassium ferricyanide and potassium ferrocyanide were used to observe and monitor the pitting corrosion of 304 stainless steel (SS) at anodic polarization in situ. The experiment verified the presence of Fe^3+^ around the pits of 304 stainless steel in 3.5% NaCl solution.

2. The Fe^3+^ around the pits could significantly reduce the pH value of the surrounding solution. Meanwhile, Fe^3+^ might be involved in the cathodic process of the corrosion for 304 SS. The reduction of Fe^3+^ leads to the positive shift of the corrosion potential of the material, which may lead to the difficulty of the hydrogen evolution reaction. The decrease of solution pH and the reduction of Fe^3+^ resulted in the rupture of passive film of 304 SS and the acceleration of the corrosion rate.

3. During the pitting process of stainless steel, especially in the metastable pitting stage, in addition to oxygen and hydrogen reduction, there is also Fe(III) reduction reaction outside the pits. Considering the pathway of Fe(III) production, we made bold assumptions: Fe^2+^ generated by metal dissolution forms Fe^3+^ under the action of dissolved oxygen, and then be reduced to Fe^2+^ on the electrode surface. In this process, dissolved oxygen does not directly participate in the cathode reaction, but indirectly participates in the cathode reaction by oxidizing Fe^2+^ to Fe^3+^. If this hypothesis is confirmed, it will have a significant impact on the study of the pitting corrosion mechanism. 

## Figures and Tables

**Table 1:** The iron ion solution with different Fe^3+^/Fe^2+^ ratios for electrochemical testing.

Composition	Solutions			
	0% Fe^3+^	10% Fe^3+^	50% Fe^3+^	100% Fe^3+^
FeCl_2_ (mol/L)	0.10	0.09	0.05	0
FeCl_3_ (mol/L)	0	0.01	0.05	0.10

**Table 2:** Fitting values for polarization curves of 304 SS in 0.1 mol/L iron ion solution with different proportion of Fe^3+^.

Testing Conditions	*E*_corr_/mV	*J_corr_*/μA·cm^−2^
0.1 mol/FeCl_2_	−0.316	1.09
0.09 mol/FeCl_2_ + 0.01 mol/FeCl_3_	−0.174	0.98
0.07 mol/FeCl_2_ + 0.03 mol/FeCl_3_	0.003	2.68
0.05 mol/FeCl_2_ + 0.05 mol/FeCl_3_	0.069	2.95
0.1 mol/FeCl_3_	0.123	3.42

**Table 3:** Equilibrium electrode potential of cathodic reduction and self-corrosion potential of 304 stainless steel in 0.1 mol/L iron ion solution with different proportion of Fe^2+^/Fe^3+^.

Potential V/SCE	Solutions				
	0% Fe^3+^	10% Fe^3+^	30% Fe^3+^	50% Fe^3+^	100% Fe^3+^
$E_{O}^{e}$	0.786 ± 0.012	0.849 ± 0.006	0.868 ± 0.003	0.878 ± 0.003	0.888 ± 0.003
$E_{H}^{e}$	−0.443 ± 0.012	−0.380 ± 0.006	−0.361 ± 0.003	−0.351 ± 0.003	−0.341 ± 0.003
$E_{Fe^{2+}/Fe^{3+}}^{e}$	0.232	0.471	0.505	0.527	0.823
*E_corr_*	−0.316	−0.174	0.003	0.069	0.123
